# Early signal detection of adverse events following influenza vaccination using proportional reporting ratio, Victoria, Australia

**DOI:** 10.1371/journal.pone.0224702

**Published:** 2019-11-01

**Authors:** Hazel J. Clothier, Jock Lawrie, Melissa A. Russell, Heath Kelly, Jim P. Buttery

**Affiliations:** 1 Monash Centre for Health Research Implementation, Monash University, Clayton, Australia; 2 SAEFVIC, Murdoch Children’s Research Institute, Parkville, Victoria, Australia; 3 School of Population & Global Health, Melbourne University, Parkville, Victoria, Australia; 4 School of Population Health, Australian National University, Canberra, Australia; 5 Ritchie Centre, Hudson Institute, Monash Health, Clayton, Victoria, Australia; 6 Monash Immunisation, Monash Health, Clayton, Victoria, Australia; The University of Hong Kong, CHINA

## Abstract

**Introduction:**

Timely adverse event following immunisation (AEFI) signal event detection is essential to minimise further vaccinees receiving unsafe vaccines. We explored the proportional reporting ratio (PRR) ability to detect two known signal events with influenza vaccines with the aim of providing a model for prospective routine signal detection and improving vaccine safety surveillance in Australia.

**Methods:**

Passive AEFI surveillance reports from 2008–2017 relating to influenza vaccines were accessed from the Australian SAEFVIC (Victoria) database. Proportional reporting ratios were calculated for two vaccine-event categories; fever and allergic AEFI. Signal detection sensitivity for two known signal events were determined using weekly data; cumulative data by individual year and; cumulative for all previous years. Signal event thresholds of PRR ≥2 and Chi-square ≥4 were applied.

**Results:**

PRR provided sensitive signal detection when calculated cumulatively by individual year or by all previous years. Known signal events were detected 15 and 11 days earlier than traditional methods used at the time of the actual events.

**Conclusion:**

Utilising a single jurisdiction’s data, PRR improved vaccine pharmacovigilance and showed the potential to detect important safety signals much earlier than previously. It has potential to maximise immunisation safety in Australia. This study progresses the necessary work to establish national cohesion for passive surveillance signal detection and strengthen routine Australian vaccine pharmacovigilance.

## Introduction

Criticism of adverse event following immunisation (AEFI) signal event detection in Australia has included that detection occurred relatively late, unnecessarily exposing further Australians to unsafe vaccines [1]. Typically, signals have been detected only following a reporter or surveillance team member describing an anecdotal impression of a change in AEFI presentations or reports [2].

The most notable event occurred in 2010 when increased incidence of fever and febrile seizures occurred following seasonal influenza vaccines [3, 4]. This was subsequently identified as arising from increased reactogenicity of one tri-valent influenza vaccine brand (Fluvax^**®**^ bioCSL). The initial alert was made by Western Australia emergency department physicians. The vaccine was withdrawn 10 days later, but not before additional cases had occurred, including a prolonged febrile seizure resulting in profound disability in a previously healthy 11-month-old child [5]. The multi-million dollar compensation decision recognized the delays in reporting and state and federal response processes that were identified in an independent enquiry [1, 5].

A separate signal event occurred in 2015 when retrospective analyses of AEFI with seasonal influenza vaccines identified a probable increase in allergy-related AEFI [6]. In this instance, severity was fortunately insufficient to warrant modification of the program. Nevertheless, it highlighted continuing insensitivity of the Australian passive surveillance systems to detect AEFI signal events in a timely manner [2]. The welcome introduction of active surveillance systems has helped, however these can be costly, time consuming and usually focus on known potential risks (new vaccines, or high risk groups) [7]. The backbone of AEFI surveillance remains passive systems and it is critical they be utilised optimally for timely signal detection.

A “signal” can be defined as incidence of AEFI occurring at a higher level than is normally expected. Signal detection in vaccine vigilance requires a multi-faceted approach as AEFI range from a rare occurrence of a severe AEFI to increased incidence or increased severity of a known, often frequently occurring, AEFI [8, 9]. While rare but particularly serious events can be detected through review of each individual report or active surveillance, an increased incidence in a more common AEFI is often more difficult to detect [10, 11], and has been described as akin to “finding a needle in the haystack” [12].

If there is known high potential for a change in AEFI incidence, as demonstrated with febrile and allergy-related AEFI related to seasonal influenza vaccines [6, 13, 14], the extra resources and intensity of active surveillance may be warranted [7]. However, for unanticipated changes in reporting, passive AEFI surveillance can provide effective means of signal detection if routine monitoring systems are applied [9]. A benefit of passive surveillance is the lower resource intensity and the stability of routine systems. The best signal detection methodologies will be those that have equal low resource requirements, do not require extensive computational skills and are easy to interpret [15].

Statistical methods for pharmacovigilance signal detection are well established but their performance characteristics remain incompletely defined [9, 16, 17]. While there are various justifications for the benefits of individual methods [18–22], no single approach is optimal in all situations [23–25] and a range of frequentist and / or Bayesian measures of disproportionality are used internationally [9, 16, 19, 26]. In Australia, the national regulator Therapeutic Goods Administration (TGA) conducts bi-monthly proportional reporting ratios (PRR) for collated national AEFI surveillance data accompanied by weekly clinical review of line lists [27, 28]. However, there are no published reports on their routine use in Australia, or evaluation of their ability to detect signal events. Investigation of these signal detection tools and applicability within the resource structure of Australian systems is therefore timely.

### Proportional reporting ratio (PRR)

This analysis focusses on proportional reporting ratio as a measure of disproportionality [29, 30]; chosen because it is an established signal detection algorithm (SDA) in pharmacovigilance [31, 32] and easy and practical to employ. Moreover it was an approach recommended in the response to national review into the management of a significant adverse event [33, 34].

The PRR is defined as the ratio between the frequency with which a specific adverse event is reported for the vaccine of interest (relative to all adverse events reported for the vaccine) and the frequency with which the same adverse event is reported for all vaccines in the comparison group (relative to all adverse events for vaccines in the comparison group). Although the PRR is an established measure, the definition of the comparator group used in calculations can vary, hence it is important to evaluate its performance prior to implementation in a new dataset.

We explore the PRR SDA as a simple to use, well defined SDA on passive vaccine AEFI surveillance data from the Victorian state vaccine safety service, SAEFVIC, using influenza vaccines as an example with the aim of providing a model for using an SDA and improving pharmacovigilance in Australia.

### Objectives

To apply PRR to routine SAEFVIC surveillance data for specific AEFI with seasonal influenza vaccines and

- Determine the time period for data calculation sufficient to meet case count requirements
- Determine ability to detect known safety signals
  - 2010 fever/febrile seizures (Fluvax^**®**^ brand specific)
  - 2015 allergic reactions
- Investigate impact of vaccine brand and age subgroup analyses on signal detection
- Ascertain generation of other potential / spurious signals

## Methods

SAEFVIC is approved under the auspices of the Human Research Ethics Committee, Royal Childrens Hospital, Victoria to manage the clinical and public health surveillance database in compliance with national and jurisdictional privacy legislation (No. 37194). Data for this study were used within the public health surveillance functions for which it is approved. No patient identifyers were included in the extraction fields to generate the PRR.

### Data source

Data from AEFI reports submitted to SAEFVIC database from January 2008 to December 2017 (as at 4 April 2018) were extracted by reported date, imported and analysed in Microsoft Power BI (Version 2.56.5023.2021, March 2018) [35].

Annual reporting periods for calendar week 10 to 40 were chosen to coincide with seasonal influenza vaccine program, although it was noted that in 2015, week 16 was the first week of reporting due to late commencement of the seasonal vaccination program [36].

### Seasonal influenza vaccines

In Australia annual influenza vaccination is recommended for anyone six months of age and older, with persons aged 65 years and over, pregnant women and those who suffer chronic conditions eligible for free vaccine under the National Immunisation Program [37]. Seasonal influenza vaccines have changed across the years and may be funded differently for different target groups (e.g children / health care workers). Reports for all seasonal influenza vaccines were combined for analyses, except where signal investigation of brand specific reactogenicity were performed. Where junior formulations were reported, they were grouped with the parent brand for analysis. Monovalent pandemic influenza vaccines H1N1Pdm2009 were excluded.

### AEFI reactions

AEFI reactions are categorised by SAEFVIC immunisation nurses according pre-determined case definitions based on symptom description [35]. We created two adverse event groups collating AEFI relating to fever or allergic reactions (below).

- **Fever** (fever unmeasured, fever ≥38<40, fever ≥40 and febrile seizure)
- **Allergy-related** (allergic reaction generalised, urticaria, anaphylaxis, angioedema)

### Calculations

Proportional reporting ratio were calculated according to a 2x2 table (Table 1).

**Table 1:** Vaccine and adverse event 2x2 contingency table.

	AEFI of Interest	Other AEFI	Sum
Vaccines of interest	a	b	a+b
Other vaccines	c	d	c+d
Sum	a+c	b+d	a+b+c+d

PRR = [*a*/(*a*+*b*)]/[*c*/(*c*+*d*)]

Chi-square (ChiSq) calculated as [12, 38]:

$$\chi^{2}=\frac{{\left(ad-bc\right)}^{2}(a+b+c+d-1)}{\left(a+b)(c+d)(a+c)(b+d\right)}$$

At 1 degree of freedom and a 5% error the tabulated value is 3.84.

Empirical thresholds were applied [12, 39], being:

- a minimum vaccine-event case-count (a) of 3
- signal alert threshold of PRR ≥2
- statistical significance test of ChiSq ≥4

Proportional reporting ratios were calculated for seasonal influenza vaccines with each of the adverse event-pair groups (fever and allergic reactions).

Three time period analyses were tested comparing the influenza vaccine-event ratio with the rationale that cumulative data would provide larger sample size for disproportionality assessment:

1. By week reported in that week alone
2. Cumulative by week in that individual year, and
3. Cumulative by week with all previous years data.

### Signal detection

Analyses were undertaken to ascertain if PRR would have assisted detection of known/suspected signal events (sensitivity):

1. 2010 febrile event [3]
2. 2015 allergy event [6]

### Brand subgroup analysis

If a signal was suggested (PRR ≥ 2 and ChiSq ≥4), the PRR was calculated for influenza vaccines as a specific subgroup, whereby each of that seasons individual influenza brands (as a ratio to the other influenza vaccine brands used in that year) to ascertain brand specific contribution to the increased PRR [38].

### Age subgroup analysis

Two age groups were defined as children (0–4 years) and 5 years and older reflecting the Australian National Immunisation Program [37]. PRR were calculated using single year and all previous year denominator data to ascertain impact of age subgroup on signal detection and signal magnitude.

### Past signal subanalysis

It was considered possible that inclusion of increased reporting from signal events in the cumulative all-years denominator may reduce the proportional ratio and mask a signal in subsequent years. Therefore we re-examined the data by excluding 2010 and 2015 from the respective cumulative all-year denominator analyses for fever and allergic reaction AEFI in calculations for each year following the known signal event.

### Signal hypothesis generation

For each signal investigated the number of weeks with PRR above threshold and the estimated proportion for which a signal could be confirmed or for which an investigation could/should have been initiated was calculated.

## Results

A total of 12,152 AEFI reports were accessed of which 1649 (13.6%) related to administration of a seasonal influenza vaccine (Table 2). There was higher reporting for females receiving influenza vaccines (male:female ratio 1:2.2) than with all vaccines (male:female ratio 1:1.2) reflecting the funded influenza vaccine program for healthworkers, who are predominantly female. The mean time to report was 4 days with 95% of all reports received within 6 months. Reports by influenza vaccine brand are provided in supplementary data (S1 Table).

**Table 2:** Summary of influenza vaccine AEFI reports received by SAEFVIC, Victoria 2008–2017.

Year	AEFI reports	Influenza (% of all reports)	Influenza reports with fever AEFI (% of Influenza reports)	Influenza reports with allergy-related AEFI (% of Influenza reports)
2008	843	40 (4.7%)	0 (0.0%)	4 (10.0%)
2009	1121	95 (8.5%)	7 (7.4%)	15 (15.8%)
2010	989	317 (32.1%)	217 (68.5%)	22 (6.9%)
2011	1091	178 (16.3%)	21 (11.8%)	21 (11.8%)
2012	887	108 (12.2%)	12 (11.1%)	12 (11.1%)
2013	1200	132 (11.0%)	31 (23.5%)	19 (14.4%)
2014	1327	140 (10.6%)	29 (20.7%)	16 (11.4%)
2015	1406	163 (11.6%)	17 (16.6%)	36 (22.1%)
2016	1451	216 (14.9%)	26 (12.0%)	26 (12.0%)
2017	1837	260 (14.2%)	28 (10.8%)	20 (7.7%)
**Total**	**12152**	**1649 (13.6%)**	**398 (24.1%)**	**191 (11.6%)**

### PRR calculation by time period

#### Reporting week

Due to low case numbers reported in any one week, the threshold of a ≥3 was reached irregularly and in some years not at all, resulting in inconsistent reporting of PRR for both fever and allergic AEFI categories. We therefore progressed to cumulative analyses.

#### Cumulative by week and individual year

**Fig 1:**
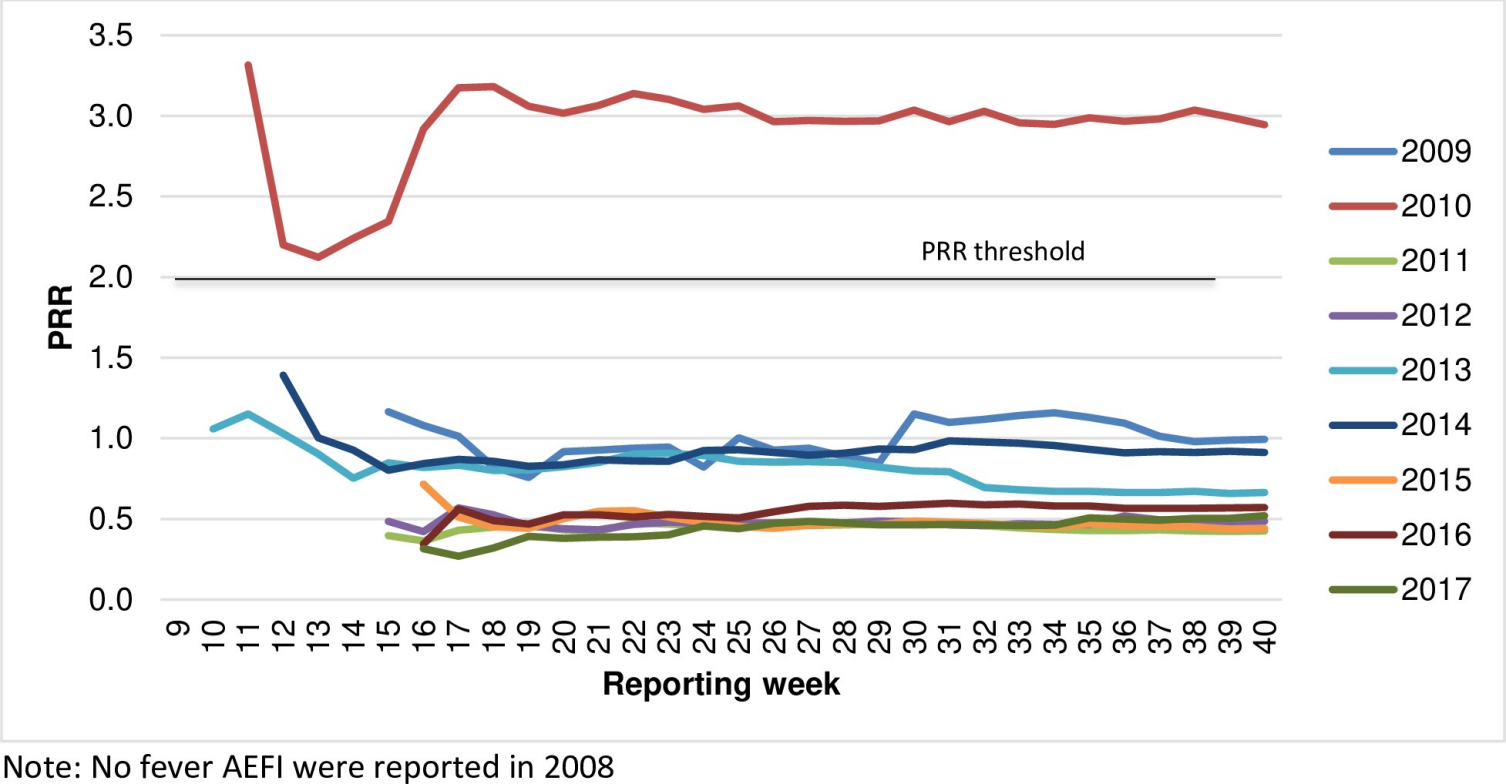
PRR cumulative (individual year) for Influenza vaccines and fever AEFI, by reported year and week, 2008–2017.

Monitoring of data cumulatively in each year provided case numbers consistently above the calculation threshold (a ≥3) for PRR calculation. For Influenza vaccines and fever reactions, a PRR ≥2 was attained only in 2010 when in fact the PRR was >2 and ChiSq >4 in all of the 17 weeks of reporting (Fig 1).

**Fig 2:**
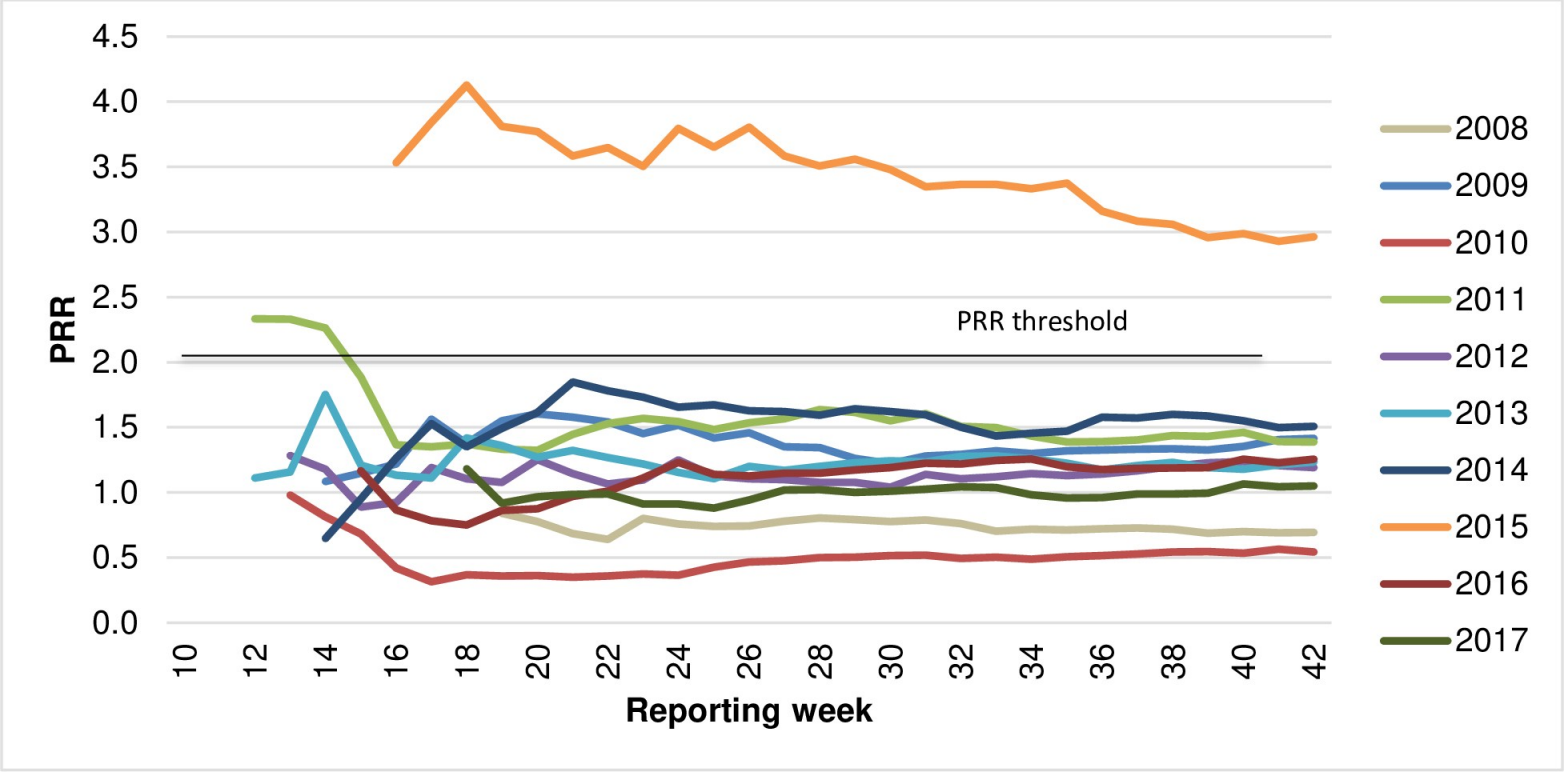
PRR cumulative (individual year) for Influenza vaccines and allergy-related AEFI, by reported year and week, 2008–2017.

For influenza vaccines and allergic reactions, signal threshold of PRR ≥2 and ChiSq ≥4 was attained in the first two weeks of reporting in 2011 before subsiding below threshold level as the number of reports received increased. Signal thresholds were reached consistently across all reporting weeks in 2015 (Fig 2).

#### Cumulative by week and all previous years data

**Fig 3:**
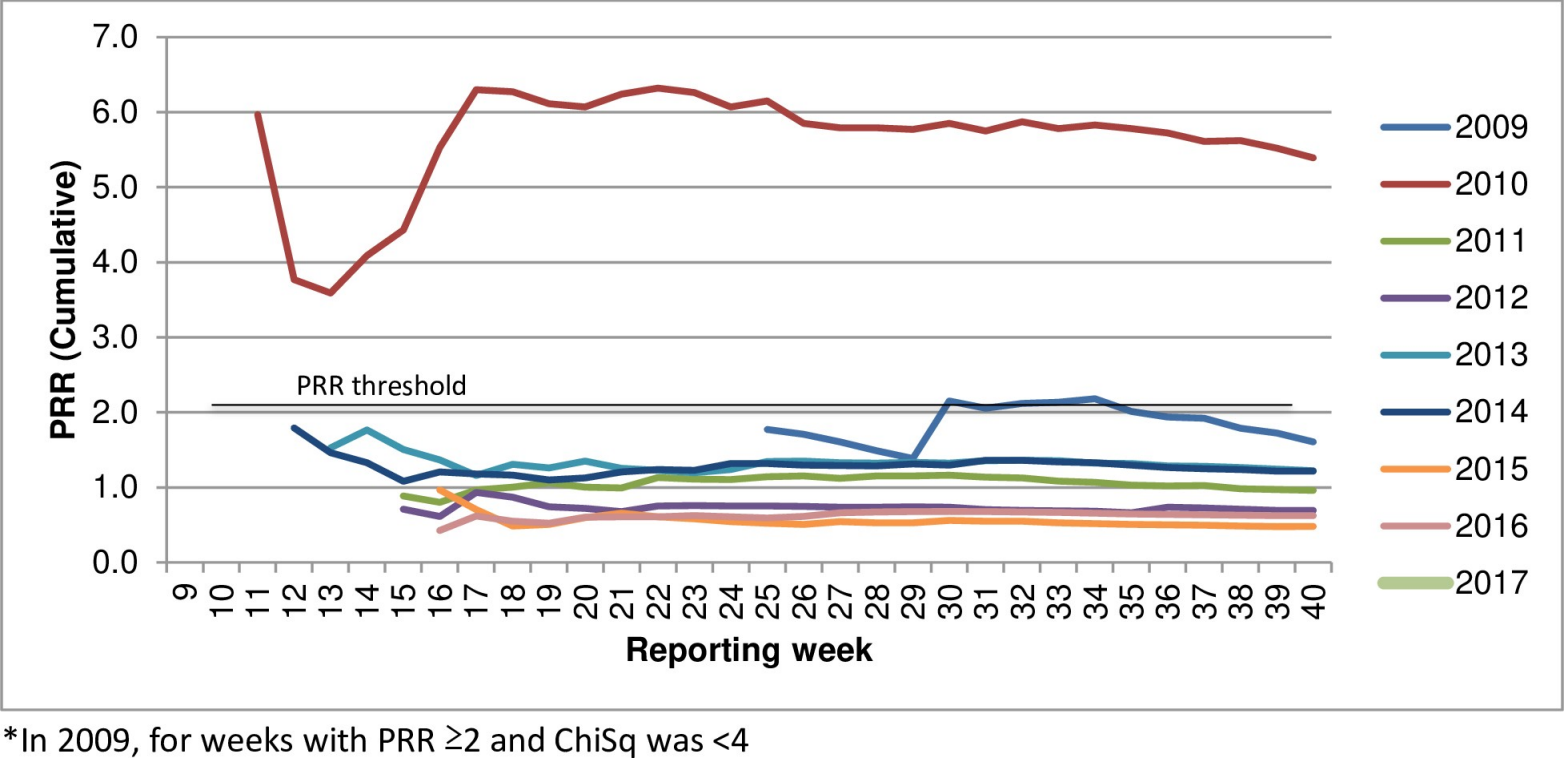
PRR cumulative (all previous years) for influenza vaccines & fever, by year.

**Fig 4:**
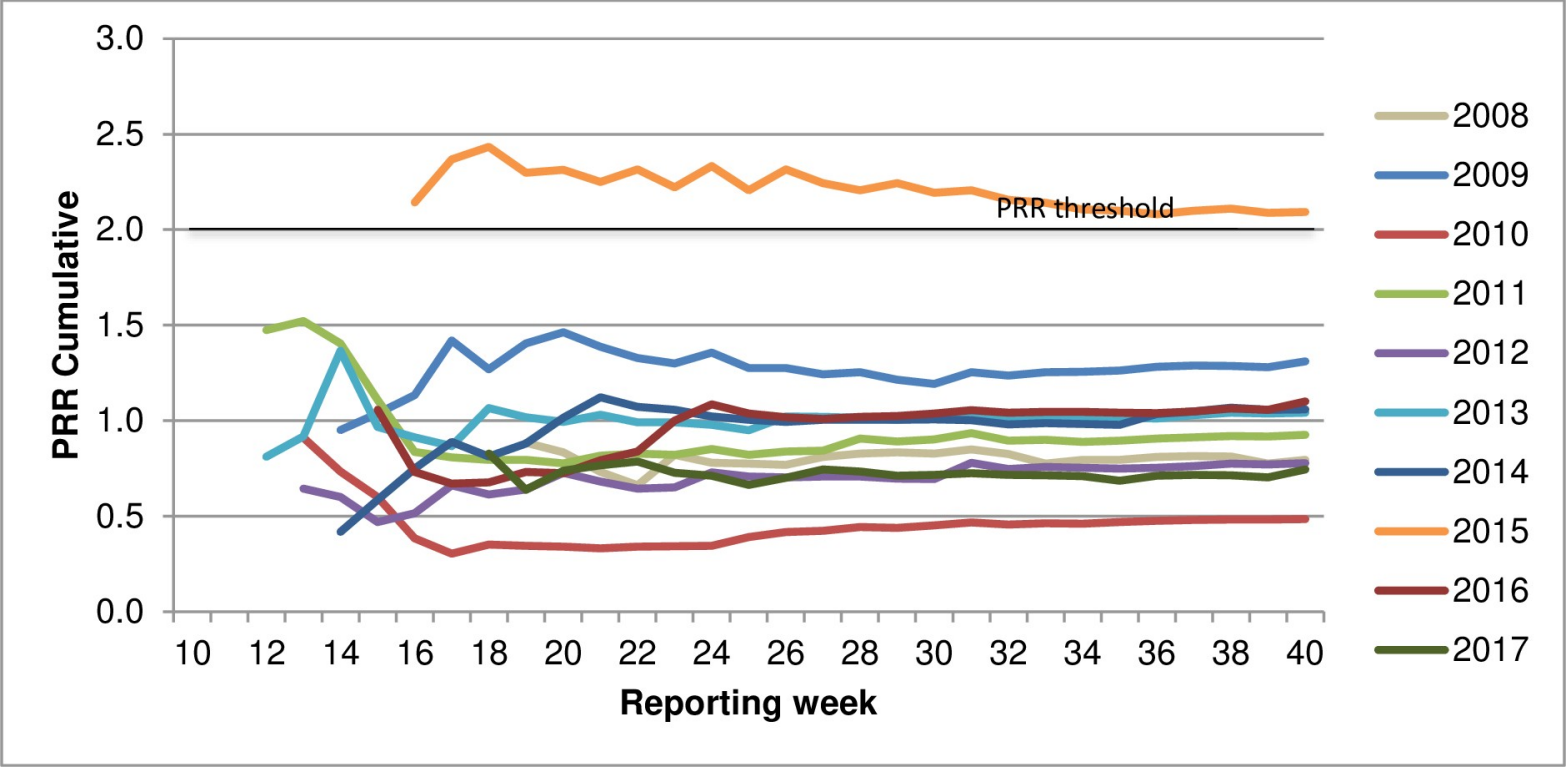
PRR cumulative (all previous years) for influenza vaccines & allergy, by year.

Both signals were detected using cumulative data for all years available (i.e., proportion of all other vaccines with the AEFI of interest in all previous years to the same reporting week time point) (Figs 3 and 4).

A comparison of PRR and ChiSq calculated using single year data or using all previous years data demonstrated variable effect on the signal magnitude (Table 3). When using all-years data in the denominator compared to the single year data, the magnitude of PRR and ChiSq significance more than doubled for the 2010 signal of influenza vaccines and fever reactions but—while still above signal thresholds—almost halved for the 2015 signal of influenza vaccines and allergy AEFI.

**Table 3:** Comparison of results using single year data or all previous year data for 2010 and 2015 signal detection^a^.

Reporting week^a^	2010 Febrile AEFI signal				2015 Allergy-related AEFI signal			
	Single year		All data		Single year		All data	
	PRR	ChiSq	PRR	ChiSq	PRR	ChiSq	PRR	ChiSq
1^st^ week	3.32	6.96	5.97	17.15	3.51	8.69	2.13	3.22
2^nd^ week	2.20	4.73	3.77	14.49	3.82	18.87	2.36	8.30
3^rd^ week	2.12	5.32	3.59	16.81	4.11	29.91	2.43	13.27
4^th^ week	2.24	8.80	4.09	29.68	3.79	33.32	2.29	15.17
5^th^ week	2.34	15.87	4.43	54.48	3.76	36.86	2.30	17.66
6^th^ week	2.92	52.31	5.53	158.18	3.57	34.30	2.25	16.65

^a^Data presented in consecutive weeks starting from commencement of reporting in that year.

(For 2010 this is calendar weeks 11–16 and in 2015 weeks 16–21)

### Sensitivity and subgroup analyses

#### 2010 fever signal

**Fig 5:**
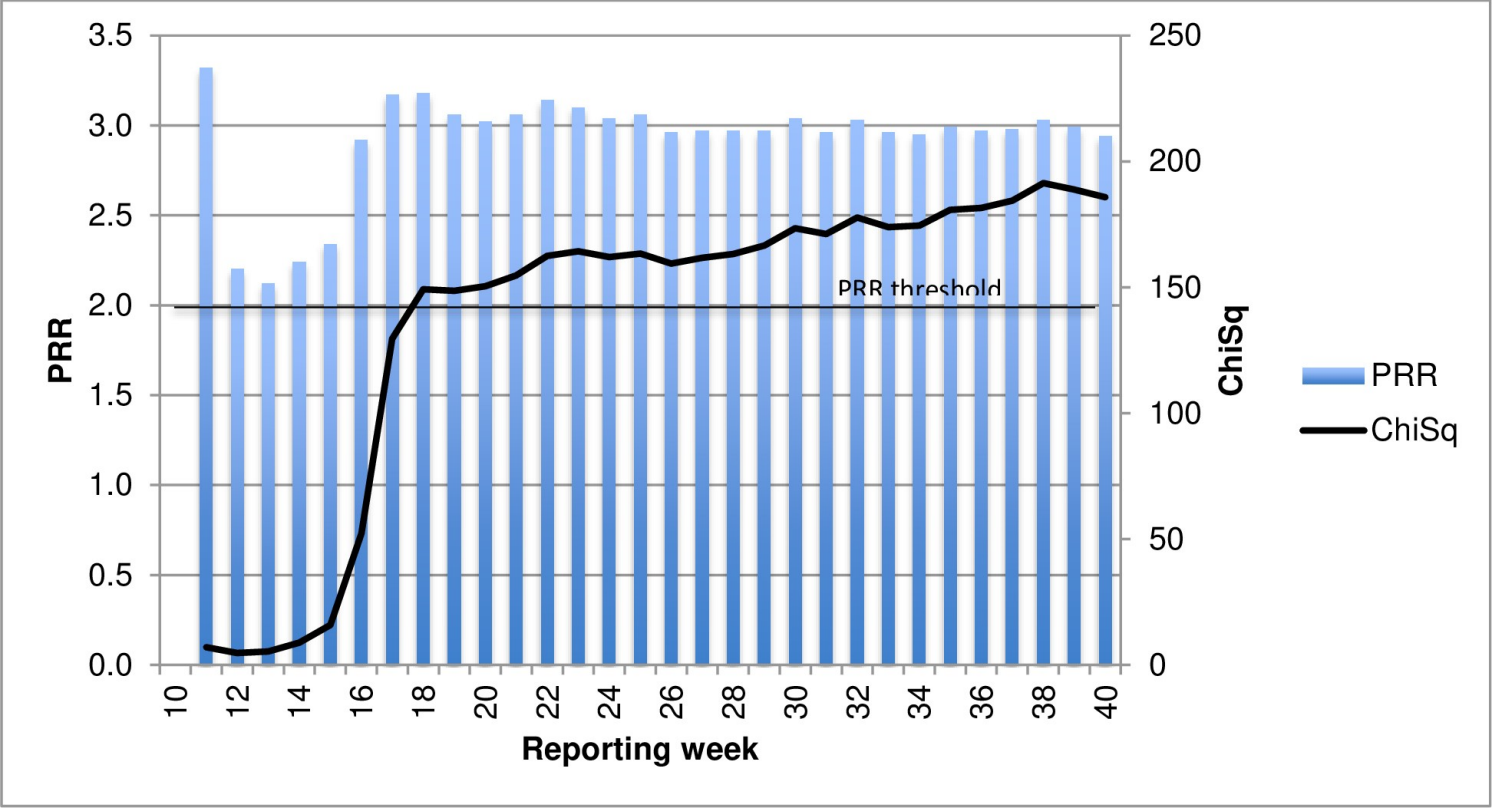
PRR cumulative and ChiSq for influenza vaccines and fever reactions by reporting week, 2010.

**Fig 6:**
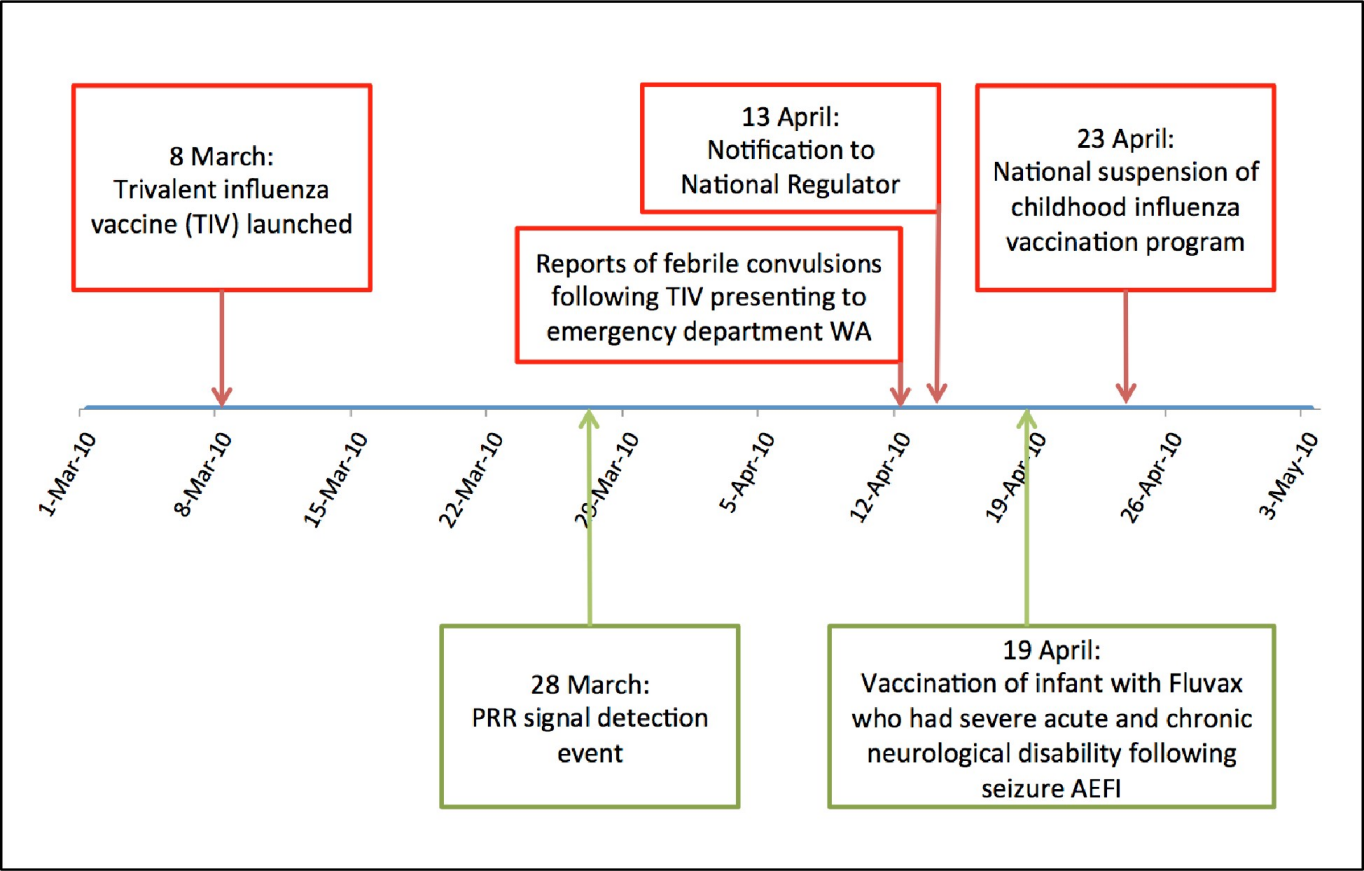
Timeline of 2010 influenza vaccine and fever AEFI signal detection.

In 2010 a signal would reasonably have been indicated by at least week 13 (using single year data; likely earlier with cumulative all-years data), which equates to 28 March 2010. At this time point the PRR = 2.12 with ChiSq 5.32 (Fig 5). This was 15 days earlier than the alert raised through clinical observation (12 April) and 26 days before the child influenza program was suspended nationally (23 April) (Fig 6).

**Fig 7:**
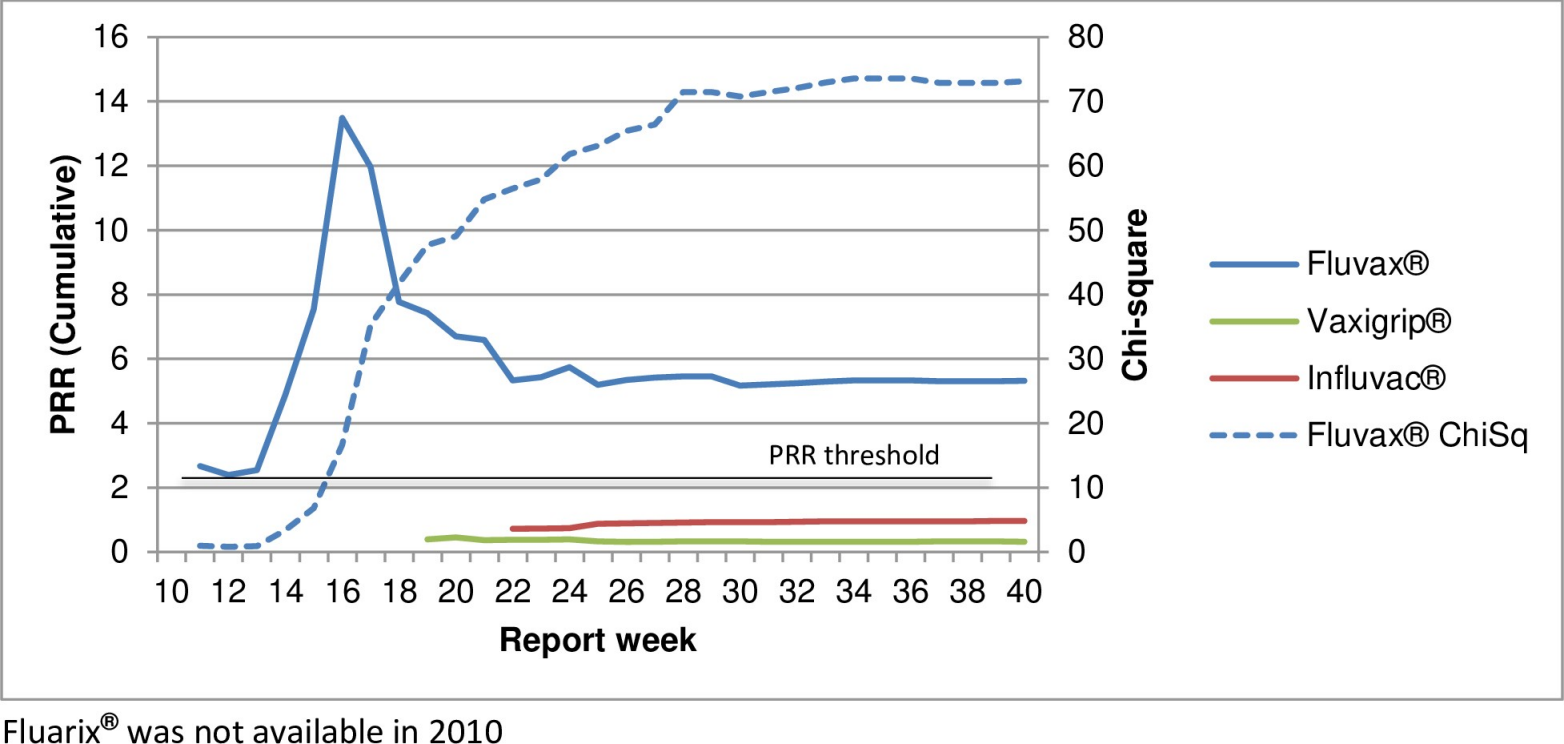
PRR by influenza brand to other infleunza vaccines, fever AEFI, 2010.

Sub-analysis comparing individual influenza vaccine brands with the other brands, for fever AEFI in 2010 shows that the proportional reporting of fever with Fluvax^**®**^ was significantly higher than the other brands in use that year (Fig 7).

Sub-analysis by age group using single year data brought higher magnitude (PRR 5.74, ChiSq 15.88) and earlier detection (calendar week 12, March 21, 2010) in the 0–4 age group. However, case count threshold was not reached for PRR calculation in 2008 and 2009 and was delayed in the remaining years by between 3–8 weeks compared to analyses across all ages. In the 5 years and older age subgroup signal magnitude was increased (PRR = 3.28, ChiSq 13.22) but was delayed by 2 weeks till calendar week 15 (first week calculable). The increased magnitude was more pronounced using cumulative all previous year data PRR 8.72, ChiSq 24.15, however the 2 week delay remained.

#### 2015 allergy signal

**Fig 8:**
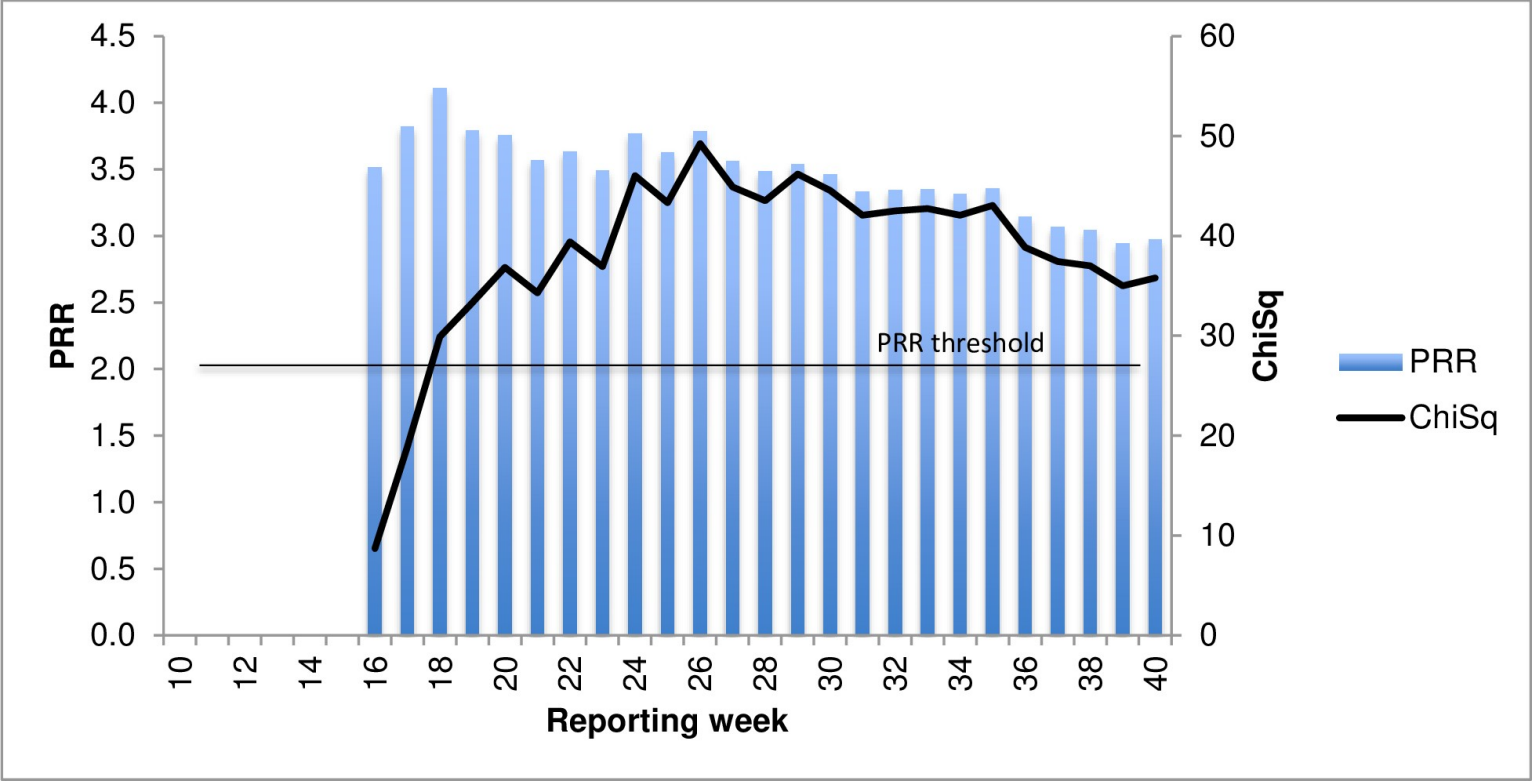
PRR cumulative and ChiSq for influenza vaccines and allergic reactions by reporting week, 2015.

In 2015 the PRR was consistently >2 from onset of reporting, with ChiSq reaching >4 (8.2) in week 16 (PRR 3.51) which equates to April 19 2015. This was 11 days earlier than the alert raised through clinical observation (1 May) (Fig 8).

Sub-analysis by age group did not facilitate signal detection in 2015. Case count in the 0–4 year age subgroup reached threshold for calculation in only three of the 10 years under investigation (2010, 2012 and 2016) and not at all in the signal event year of 2015. Signal threshold was reached in 2015 for the 5 years and older age subgroup, but one week later and at a lower magnitude (PRR = 2.48) compared to analysis across all ages. Cumulative analysis using all previous year data did not reach signal detection thresholds in either age subgroup.

**Fig 9:**
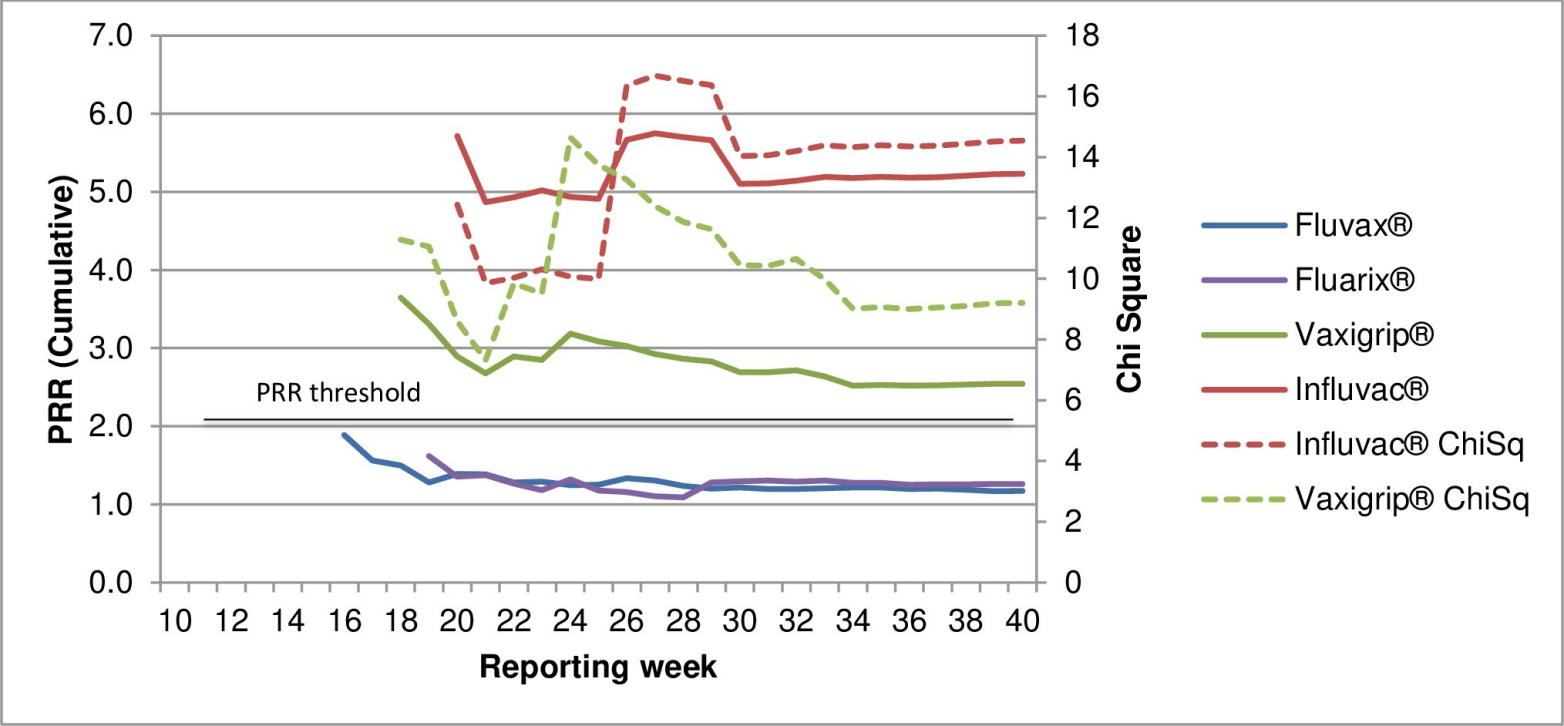
PRR by influenza vaccine brand to other brands, allergic AEFI, 2015.

Sub-analysis comparing individual influenza vaccine brands with the other brands, for allergic AEFI in 2015 shows that the proportional reporting of allergic AEFI with Vaxigrip^**®**^, Sanofi Pasteur and Influvac^**®**^, Solvay Pharmaceuticals were significantly higher than for Fluvax^**®**^ and Fluarix^**®**^, GlaxoSmithKline that year (Fig 9).

Sub-analysis removing signal year data from the all previous years cumulative denominator when calculating PRR for subsequent years did not elicit any additional signal detections.

### Signal hypothesis generation

This analysis intended to document the number of weeks in which PRR and ChiSq reached signal threshold levels of ≥2 and ≥4 respectively, and correlate the proportion that matched with known events (positive detection) as well as to indicate the proportion of weeks when an investigation could have been suggested (hypothesis generation). The data were calculated for each week and for each reporting year as single year data and as all previous years data. Signal thresholds, aside from the two known events, were reached only in the first two weeks (weeks 13 and 14) of seasonal surveillance in 2011.

The analysis for Influenza vaccines and fever AEFI was repeated for all subsequent years, excluding 2010 data, and did not produce any additional reporting weeks reaching PRR ≥2.

### Outcomes

○ PRR identified the two known signals of influenza vaccines and fever / allergic reaction
○ Statistical evidence hypothesised a signal 15 and 11 days earlier than actual alert dates respectively
○ Cumulative data period influences signal magnitude depending on AEFI association to vaccine
○ PRR sub-analysis by vaccine brand supports hypothesis of brand specific signals
○ Using PRR generated one signal hypothesis requiring further investigation for signal validation

## Discussion

This retrospective analysis demonstrated PRR as a feasible and applicable signal detection algorithm for two known influenza vaccine signal events. If cumulative PRR analysis had been in use at the time, we would have been able to identify the febrile signal in 2010 and allergy signal in 2015 much earlier than was possible by signal monitoring methods in place at those time points. Earlier signal detection and investigation implicitly enables earlier action if warranted, which in 2010 could have prevented many children receiving an unsafe vaccine. In 2015, it would have helped clarify the risk-benefit information and improve consumer confidence to continue with vaccination.

While the longevity of the SAEFVIC database with over a decade of data benefited cumulative calculations, a single year of data sufficed for effective signal detection. This is important for newly established datasets. It is also an important performance requirement if monitoring new vaccines (e.g Zostavax^**®**^ Merck vaccines), or new brands or formulations (e.g Garadsil^**®**^9 Seqirus) or population subgroups (e.g in pregnancy) as it may not be appropriate to include disparate historical AEFI reporting data in the comparator group.

There was discordant impact on efficiency of signal detection when using cumulative data from all previous years compared to data from a single year. In 2010 with fever AEFI the signal was magnified, but in 2015 with allergy AEFI the signal diminished. This may occur as fever is a common AEFI with many vaccines but allergy-related AEFI occur more frequently with influenza vaccines due to the multiple antigens and the manufacturing process being in eggs with small amounts of egg albumin retained in influenza vaccines [40]. The inclusion of higher numbers of allergy-related reports from previous years in the denominator therefore diminishes the signal.

Analysis by population or categorical subgroups (e.g age, gender or geographic area) has potential to increase sensitivity for signal detection but may also mask signals [38]. Constraints can arise through small case numbers leading to a delay in reaching calculation threshold and consequently signal detection opportunities being equally delayed. However, jurisdictional funding in recent years as well as the July 2019 Pharmaceutical Benefits Advisory Committee recommendation for seasonal influenza vaccination of children aged 6 months to 5 years to be included in the National Immunisation Program [41], is likely to boost numbers in this age group and therefore increase the utility and sensitivity of age subgroup analyses [42].

The population under surveillance is all vaccinees and when operating prospectively it will not be known in which age group a signal may arise in, therefore it is appropriate to analyse across the whole population routinely, as well as by subgroup [38].

This study hypothesises that Vaxigrip^**®**^ and Influvax^**®**^ vaccine brands flagged in the 2015 allergy-related signal event, which is in contrast to the findings in the previous investigation, which concluded there was no implication of a specific brand[6]. The previous analysis however, was limited to calculating brand-specific reporting rate per 100,000 doses distributed; the rates of which did not differ significantly between brands. Furthermore, as Influvac^**®**^ was not a Department of Health funded vaccine in that year, dose distribution data were not available and so Influvac^**®**^ could not be included in the brand-specific analyses in that study.

The paucity of spurious signals in our study should be interpreted with caution as this study was limited to a narrow scope of influenza vaccines and only two AEFI categories. Other studies indicate the norm is to find a substantial fraction of signals, albeit for which no external supporting evidence can be found, even when a highly inclusive search for such evidence is conducted[43]. Spurious signals are likely to be detected if PRR were applied to all vaccine-AEFI event pairs occurring in the database, which would be extensive. This study used calculation and signal threshold determinants with precedence from earlier studies [12, 38]. Further exploration of these arbitrary detection thresholds may be required as changes in the standard threshold for the count of drug-event combinations can result in a substantial variation in efficiency of the signal detection process. The European Medicines Agency found a threshold of five compared to a threshold of three (as we used) gave a reduction of 25% in false positive signals in return for a loss of 12% in true signals detected early [39].

Strengths for this proof-of-principle study were the decade of data available and presence of two confirmed signal events of differing magnitude contained within. Limitations included the small number of case-event pairs, particularly in non-signal years; testing on only two signals, both involving the same vaccine group (influenza vaccines) and; the absence of documented false signals within the dataset and non-generation of spurious signals meaning an inability to examine the algorithm performance with false-positive signals. The potential for prospective analyses to inform signal detection must also be viewed commensurate with known limitations of passive surveillance systems: namely under-reporting—particularly of reactions perceived as mild—and the time lag from symptom onset to report submission. However, it is worth noting that in this study, simulating reality as data were extracted according to date reported, the safety signal was still generated well ahead of the historical clinical recognition.

To mature signal hypothesis generation, we plan to integrate PRR as a routine prospective signal detection platform with automated calculations updated weekly providing an “alert” table presenting any vaccine-event pairs reaching the pre-defined threshold and statistical significance levels for epidemiological review. This will provide signal hypothesis generation as a first step in comprehensive signal management process. Even though this will be performed on the SAEFVIC Victorian state-level data it has relevance for Australian vaccine safety nationally as SAEFVIC contributes approximately 45 per cent of all AEFI reporting to the national AEFI database [44].

Further studies to incorporate and compare performance characteristics with Bayesian algorithms and multivariate modeling techniques are required [9, 45], however the final choice of SDA should be made in the context of meeting the Innovative Medicines Initiative PROTECT (Pharmacoepidemiological Research on Outcomes of Therapeutics by a European ConsorTium) project recommendation that “*Choice of a disproportionality statistic for signal detection should be primarily based on ease of implementation*, *interpretation and optimisation of resources*” [15].

## Conclusion

The PRR algorithm is relatively easy to implement and analyse. Utilising a single jurisdiction’s data, PRR identified a loud (febrile) and a quieter (allergy) signal in the SAEFVIC data set. There is no reason to think it would not be as successful prospectively as it has been shown to be retrospectively. We believe the PRR should be routine in SAEFVIC and in any national adverse event surveillance system that evolves.

The implementation of signal detection algorithms in routine Australian vaccine pharmacovigilance has the potential to detect important safety signals much earlier than previously, keeping immunisations in Australia as safe as possible and helping maintain community and provider confidence.

## Supporting information

S1 Table AEFI reports by Influenza vaccine brand and year reported, 2008–2017, SAEFVIC. (DOCX)
