# Supplementary material for: Early signal detection of adverse events following influenza vaccination using proportional reporting ratio, Victoria, Australia
Source: PLoS One. 2019 Nov 1;14(11):e0224702. doi: 10.1371/journal.pone.0224702 (PMC6824574; doi:10.1371/journal.pone.0224702)
Supplement: S1 Table — (DOCX) [file pone.0224702.s001.docx]

Table S1: AEFI reports by Influenza vaccine brand and year reported, 2008-2017, SAEFVIC

| **Year reported** | **Influenza vaccine brand (% of influenza AEFI reports in that year)** | | | | | | | | | **Total** |
| --- | --- | --- | --- | --- | --- | --- | --- | --- | --- | --- |
|  | **Afluria Quad®** | **Fluarix®** | **Fluarix Tetra®** | **FluQuadri®** | **Fluvax®** | **Influvac®** | **Intanza®** | **Vaxigrip®** | **Brand unknown** |  |
| **2008** |  |  |  |  | 17 (45.5%) | 3 (7.5%) |  | 19 (47.5%) |  | 40 |
| **2009** |  |  |  |  | 51 (53.7%) | 4 (4.2%) |  | 41 (43.2%) |  | 95 |
| **2010** |  |  |  |  | 264 (83.3%) | 11 (3.5%) | 4 (1.3%) | 38 (12.0%) |  | 317 |
| **2011** |  | 1 (0.6%) |  |  | 57 (32.0%) | 53 (29.8%) | 1 (0.6%) | 66 (37.1%) |  | 178 |
| **2012** |  | 27 (25.0%) |  |  | 50 (46.3% | 7 (6.5%) | 2 (1.9%) | 15 (13.9%) | 7 (6.5%) | 108 |
| **2013** |  | 36 (27.3%) |  |  | 49 (37.1%) | 12 (9.1%) | 1 (0.8%) | 27 (20.5%) | 7 (5.3%) | 132 |
| **2014** |  | 27 (19.3%) |  |  | 57 (40.7%) | 5 (3.6%) |  | 42 (30.0) | 9 (6.4%) | 140 |
| **2015** |  | 39 (23.9%) |  | 1 (0.6%) | 45 (27.6%) | 9 (5.5%) |  | 49 (30.1%) | 20 (12.3%) | 163 |
| **2016** |  | 4 (1.9%) | 50 (32.1%) | 136 (63.0%) | 14 (6.5%) | 1 (0.5%) |  | 1 (0.5%) | 9 (4.2%) | 216 |
| **2017** | 71 (27.3%) |  | 50 (19.2%) | 111 (42.7%) | 3 (1.2%) | 2 (0.8%) |  | 3 (1.2%) | 20 (7.7%) | 260 |
